# Evidence of language-related left hypofrontality in Major Depression: An EEG Beta band study

**DOI:** 10.1038/s41598-020-65168-w

**Published:** 2020-05-18

**Authors:** Chiara Spironelli, Antonio Maffei, Zaira Romeo, Giulia Piazzon, Giordano Padovan, Gianna Magnolfi, Ilenia Pasini, Francesca Gomez Homen, Graziano Concari, Alessandro Angrilli

**Affiliations:** 1 Department of General Psychology, University of Padova, Padova, Italy; 2 Padova Neuroscience Center, University of Padova, Padova, Italy; 3 IRCCS San Camillo Hospital, Venice, Italy; 4 Psychiatric Clinic, Neuroscience Department, University of Padova, Padova, Italy; 5 ULSS 9, Bussolengo, Verona Italy; 6 CNR Institute of Neuroscience, Padova, Italy

**Keywords:** Language, Human behaviour, Depression

## Abstract

Major depression (MDD) has been associated with an altered EEG frontal asymmetry measured in resting state; nevertheless, this association has showed a weak consistency across studies. In the present study, which starts from an evolutionistic view of psychiatric disorders, we investigated frontal asymmetry in MDD, using language as a probe to test the integrity of large inter- and intra-hemispheric networks and processes. Thirty MDD patients (22 women) and 32 matched controls (HC) were recruited for an EEG recording in resting state and during two linguistic tasks, phonological and semantic. Normalized alpha and beta EEG spectral bands were measured across all three conditions in the two groups. EEG alpha amplitude showed no hemispheric asymmetry, regardless of group, both at rest and during linguistic tasks. During resting state, analysis of EEG beta revealed a lack of hemispheric asymmetry in both groups, but during linguistic tasks, HC exhibited the typical greater left frontal beta activation, whereas MDD patients showed a lack of frontal asymmetry and a significantly lower activation of left frontal sites. In depressed patients, positive affect was negatively correlated with depression levels and positively correlated with left frontal EEG beta amplitude. Language represents the human process that requires the largest level of integration between and within the hemispheres; thus, language asymmetry was a valid probe to test the left frontal alteration encompassing highly impairing psychiatric disorders, such as schizophrenia and MDD. Indeed, these severe diseases are marked by delusions, ruminations, thought disorders, and hallucinations, all of which have a clear linguistic or metalinguistic basis.

## Introduction

A consistent literature has investigated abnormal brain organization and functioning in major depressive disorder (MDD) patients^1–3^. Evidence from functional and metabolic neuroimaging techniques reveals that MDD patients reliably show reduced brain activity in frontal areas during waking, a phenomenon known as hypofrontality, i.e., a significant dysfunction in the activation of the prefrontal cortex^2^, typically associated with deficits in attention, action planning, working memory and sleep (Siddiqui *et al*., 2008)^4^. Given that prefrontal cortex function has repeatedly been implicated in the implementation of cognitive control^5^, MDD patients’ hypofrontality may relate to their difficulty in organizing behavior to conflict resolution.

MDD patients’ hypofrontality has also consistently been found – especially in the left hemisphere – with electrophysiological techniques analyzing slow EEG activity during resting state^6–8^. Among other low frequency EEG rhythms, the alpha band (8–12 Hz) has been considered a reliable marker of decreased cortical arousal not only during sleep, but also in adult individuals who are awake but not engaged in specific cognitive tasks^9,10^. With particular regard to resting state frontal asymmetry, most EEG studies found increased levels of alpha rhythm in left vs. right sites of MDD patients compared with healthy controls, independent of closed/open eye condition^6,11–13^. Notwithstanding a substantial amount of literature supporting this hypothesis, some studies failed to confirm a direct association between left frontal hypoactivity and MDD, probably due to methodological differences in data collection as well as to patients’ heterogeneity^14–16^. A review of studies focused on EEG alpha asymmetry (in resting state) as a marker of MDD found an overall null size effect and suggested that the large observed across-studies heterogeneity in frontal asymmetry must take into account also other critical variables^17^. This result clearly challenges the idea that frontal alpha asymmetry alone represents a simple straightforward diagnostic marker of depression.

According with Stewart and co-worker^13^, in addition to the measure of EEG asymmetry during resting state, it is also important to measure frontal brain asymmetries during active tasks. This decreases within-subject variability during a free non-controlled state of the patient, but it also recruits potentially altered networks reliably engaged by the task. Among others, emotional tasks have been used to test patients’ motivation and therefore modulate their frontal brain asymmetry to a greater extent than a resting state does^18^. Indeed, in a study in which participants had to recognize manipulated facial expressions, a reliable change in EEG asymmetries was found in healthy participants^19,20^ as well as in MDD patients^13,21^. This evidence clearly highlights the important role of left frontal brain regions in an active task involving emotional processing. Furthermore, poor cognitive performance is frequently observed in depressed patients, which has potential implications for the response to intervention^22–24^. Neuropsychological studies on MDD patients have highlighted that these cognitive impairments span several cognitive domains, most notably: executive function^25^, memory^26^, processing speed^27^, and attention^28^. Harvey and colleagues^25^ found deficits in executive functions (especially in updating, shifting, and inhibition processes) in young depressed in-patients. Impaired performance on the n-back task was more severe in patients with multiple hospitalizations than in those hospitalized for the first time. Moreover, major depression and schizophrenia patients showed worse performance, compared to controls, in semantic fluency, visuospatial backward and digit span forward tests^26^. Impaired performance in MDD was also found in processing speed and working memory tasks^27^. Notably, neuropsychological dysfunction in MDD appeared to be influenced by resource decrement, which also persists after remission of the depression in geriatric patients. With respect to the association between cognitive deficit and depression progress, Majer and collaborators^28^ found pathological performance in attention and executive tasks at admission, with marginal improvements at discharge; moreover, a meta-analysis focused on first-episode MDD patients revealed that cognitive impairments in attention, memory and executive functions are consistently observed since the disease onset^29^. Overall, these findings outline that cognitive deficits represent a core feature of depressive disorders^30^, related to distinctive patterns of abnormal activity in the frontal regions of the brain^31^. Keeping in mind the results from the quoted literature, the present research aimed to study hemispheric asymmetry in MDD patients from a different perspective. We started from the idea that the main aspect that is shared by major depression, bipolar disorder and schizophrenia psychiatric triad is the impairment in the cognitive domain. First, we were interested to test a specific heuristic hypothesis on a possible endophenotype, namely, altered language hemispheric asymmetry, common to all severe psychiatric disorders. At this early stage of the investigation, we did not yet aim to devise a psychophysiological marker of depression (or psychiatric severity). Recent advances in the neurobiological and genetic characterization of psychiatric diseases support the idea of a continuum of severity across the psychiatric triad^32^, which has also been included in the most recent version of the *Diagnostic and Statistical Manual of Mental Disorders* (DSM-5)^33^ and has been proposed as the main core of the Research Domain Criteria (RDoC) approach to psychoses^34^. Second, the specific hypothesis on an altered language asymmetry in MDD can be tested by means of a linguistic paradigm we have used in a number of past studies on brain plasticity of language in dyslexic children after phonological training^35^, in aphasic patients after recovery^36^ and in schizophrenia patients^37–40^. As language represents the most complex emerging property of a large and complex brain, and recruits virtually the whole cortex, any disorder that affects the delicate balance in the activation between and within the hemispheres can, in principle, alter this equilibrium and, at the same time, this alteration can explain the many symptoms and metalinguistic impaiments known to be involved in the most severe psychiatric disorders (e.g., semantic anomalies, thought disorders, ruminations, auditory hallucinations). In past studies on schizophrenia, we successfully tested Crow’s etiological hypothesis^41,42^ on the main mechanism postulated at the origin of schizophrenia (i.e., the disruption of linguistic left hemispheric dominance typically observed in healthy individuals). We also found, in the patients, a significant correlation between left frontal inhibition during active linguistic tasks and the extent of clinical delusions^40^ and hallucinations^38^, which suggested a more (hallucinations) or less (delusions) direct link between altered percepts, language and thoughts. In line with Crow’s hypothesis on the common origin of psychoses, we expected that alteration of language frontal asymmetry may also be found in other diseases of the psychiatric triad, such as MDD.

## Results

### Patients’ overview

The psychiatric evaluation classified all patients as suffering from major depressive disorder, 7 of whom showing a single MD episode (DSM-IV-TR 296.20), and the remaining 23 recurrent MD episodes (DSM-V-TR 296.30) (Table 1). Only three patients showed psychotic symptoms at the time of the experiment, and 12 out of 30 had comorbidity disorder(s) in Axis-I (typically Dysthymia or Anxiety disorders). As expected for chronic patients, the severity of depressive symptoms measured with clinical scales was, on average, below the cut-off scores of HAM-D (cut-off = 10) and BDI-2 (cut-off = 13) inventories (Table 1), thus confirming a state of (at least partial) remission from acute major depression episodes and a good compliance with pharmacological treatment. Notwithstanding this, HAM-D scores classified 11 of them within the normal ranges (0–7 points), 13 at mild level (8–13 points) and the remaining 6 at moderate level (14–18 points) of depressive symptoms, with no cases of severe/very severe levels (19–22 and >22 points, respectively). Instead, the qualitative analysis of depressive symptoms, according to BDI-II scores, classified 20 patients with minimum depressive symptoms (0–13 points), 3 with mild (14–19 points), 2 with moderate (20–28) and 5 with severe (23–63 points) levels of MDD symptoms.

**Table 1:** Demographic and clinical characteristics of MMD patients, average scores obtained to the Italian version of Hamilton Rating Scale for Depression (HAM-D; Hamilton, 1980), Beck Depression Inventory-II (BDI-II; Beck *et al*. 1996) and State-Trait Anxiety Inventory-Y1 (STAI-Y1, State version; Spielberg *et al*. 1983), and patients’ drug treatment.

PATIENT	AGE AT ONSET (YEARS)	NUMBER OF DEPRESSIVE EPISODES	PSYCHOTIC SYMPTOMS	HAM-D	BDI-2	STAI-Y1	COMORBIDITY SCID-I	ATYPICAL ANTIPSYCHOTIC	ANTIDEPRESSANT	BENZODIAZEPINE	ANTICONVULSANT
P01	23	2	no	15	33	53	Dysthymia	Quetiapine 25 mg	Paroxetine 20 mg		
P02	30	3	no	8	9	36	Lifetime Panic Disorder		Citalopram 20 mg		Lamotrigine 100 mg
P03	35	2	no	0	6	35	none		Venlafaxine 150 mg		Lamotrigine 150 mg
P04	58	4	no	10	16	34	none		Paroxetine 20 mg		
P05	55	2	no	6	4	40	none		Escitalopram 15 mg		
P06	45	2	no	13	12	56	none		Duloxetine 60 mg	Delorazepam 10 gt	
P07	40	1	no	14	36	48	none		Duloxetine 30 mg		
P08	67	2	no	4	8	28	none		—		
P09	35	1	no	17	30	68	none		—		
P10	27	3	no	8	4	40	Lifetime Panic Disorder; Social Anxiety Disorder		Venlafaxine 150 mg		Lamotrigine 100 mg
P11	52	2	no	4	2	42	none		Citalopram 20 mg	Flurazepam 30 mg	Valproate 300 mg
P12	22	1	no	9	10	31	none		Sertraline 50 mg		
P13	32	2	no	9	9	37	Dysthymia		Escitalopram 20 mg		
P14	28	2	no	14	23	57	Dysthymia; Lifetime Panic Disorder; Social Anxiety Disorder		—		
P15	32	5	no	13	3	32	none	Quetiapine RP 200 mg	Fluoxetine 20 mg		Lamotrigine 100 mg
P16	38	2	no	17	24	49	Lifetime Panic Disorder		Sertraline 150 mg	Clonazepam 3 gtt/sera	
P17	38	2	no	11	7	26	Lifetime Panic Disorder		Fluoxetine 40 mg		
P18	50	1	no	4	10	20	none		Dulaxetine 60 mg Clomipramine 25 mg Trazodone 75 mg		Pregabalin 25 mg
P19	41	3	yes	0	4	18	none			Lorazepam 2.5 mg	
P20	49	1	no	12	4	19	none		Sertraline 25 mg		
P21	49	1	no	2	14	21	none		Escitalopram 10 mg Mirtazapine 7.5 mg		
P22	48	4	no	11	33	30	Dysthymia		Paroxetine 10 mg Fluoxetine 10 mg	Alprazolam 0.50 mg	
P23	55	10	no	12	42	39	Dysthymia; Agoraphobia	Perphenazine 2 mg	Escitalopram 20 mg Venlafaxine 150 mg	Alprazolam 1 mg	
P24	25	8	no	14	1	11	none	Aripiprazole 5 mg	Venlafaxine 150 mg Clomipramine 300 mg	Lorazepam 2.5 mg	Lamotrigine 200 mg
P25	14	5	yes	2	0	20	Lifetime Panic Disorder		Duloxetine 60 mg Nortriptyline 2.5 mg	Alprazolam 10 gt	
P26	37	1	no	11	7	12	none		Venlafaxine 75 mg/150 mg alterned with Mirtazapine 7.5 mg		
P27	11.5	4	no	6	15	30	Lifetime Panic Disorder; Agoraphobia	Quetiapine 100 mg		Clonazepam 4 mg	
P28	30	9	no	1	3	20	none	Aripiprazole 10 mg	Amitriptyline 25 mg	Clonazepam 2 mg	
P29	28	2	yes	8	0	16	none	Risperidone 1 mg			Valproate 600 mg
P30	40	3	no	2	1	16	none		Citalopram Chlorhydrate 10 gt		

Concerning drug treatment, 22 patients were treated with antidepressant drugs (i.e., venlafaxine, escitalopram, citalopram, paroxetine, duloxetine, sertraline, clomipramine, trazodone, mirtazapine, fluoxetine, nortriptyline and amitriptyline), ten with benzodiazepines (i.e., lorazepam, delorazepam, flurazepam, clonazepam and alprazolam), eight patients with anti-epileptic drugs (i.e., sodium valproate, lamotrigine and pregabalin) and six with antipsychotic drugs (i.e., aripiprazole, quetiapine, perphenazine and risperidone).

With respect to the level of state anxiety, measured by the State-Trait Anxiety Inventory-Y1 (STAI-Y1, State version) in both groups, depressive patients obtained significantly higher scores than healthy controls (32.80 ± 14.53 vs. 25.84 ± 8.10, respectively; *t*(60) = −2.35, *p* < 0.05). Considering the two dimensions of affect, the Positive And Negative Affect Scale (PANAS) subscales revealed similar levels of negative affect in depressed patients (21.93 ± 10.30) and healthy controls (21.09 ± 7.26; *t*(60) = −0.37, *ns*), but significantly lower positive affect scores in the psychiatric group (29.77 ± 7.14 vs. 33.78 ± 4.87 for patients and controls, respectively; *t*(60) = 2.60, *p* < 0.01).

Considering the levels of state anxiety (STAI-Y1) and Negative Affect (NA-PANAS), we found positive correlations with all the psychiatric scales (HAM-D: *r*(28) = 0.47, *p* = 0.008, and *r*(28) = 0.56, *p* = 0.001; BDI-2: *r*(28) = 0.59, *p* < 0.001, and *r*(28) = 0.66, *p* < 0.001, respectively); the higher the level of state anxiety and the NA-PANAS score, the more severe the depressive symptoms measured by the two psychiatric scales. By contrast, the Positive Affect, measured by the PANAS (PA-PANAS), was negatively associated with the BDI-2 scale only (*r*(28) = −0.63, *p* < 0.001); the higher the PA-PANAS scores, the lower the depressive symptoms measured by the BDI-2 inventory.

### Socio-demographical and behavioral data

Groups showed no differences in age (*t*(60) = −0.22, *ns*), gender distribution (*χ*^2^(1) = 0.43, *ns*) or handedness (*t*(60) = 1.16, *ns*), but healthy controls had significantly higher education levels compared with MDD patients (*t*(60) = 2.70, *p* = 0.009). For this reason, we preliminarily carried out ANCOVA statistics on behavioral data, by adding the education level as a covariate to the ANOVAs. However, education level interacted with neither Response Times (RTs) nor Error Rates (ERs) (*F*(1,59) = 3.50, *ns*, and *F*(1,59) = 0.28, *ns*, respectively). Therefore, we showed and discussed the results from ANOVA analyses.

RTs showed a significant main effect of task factor [*F*(1,60) = 86.90, *p* < 0.001], revealing longer RTs for the semantic task (1235.58 ± 314.93 ms) than the phonological task (970.43 ± 209.46 ms), regardless of group.

**Figure 1:**
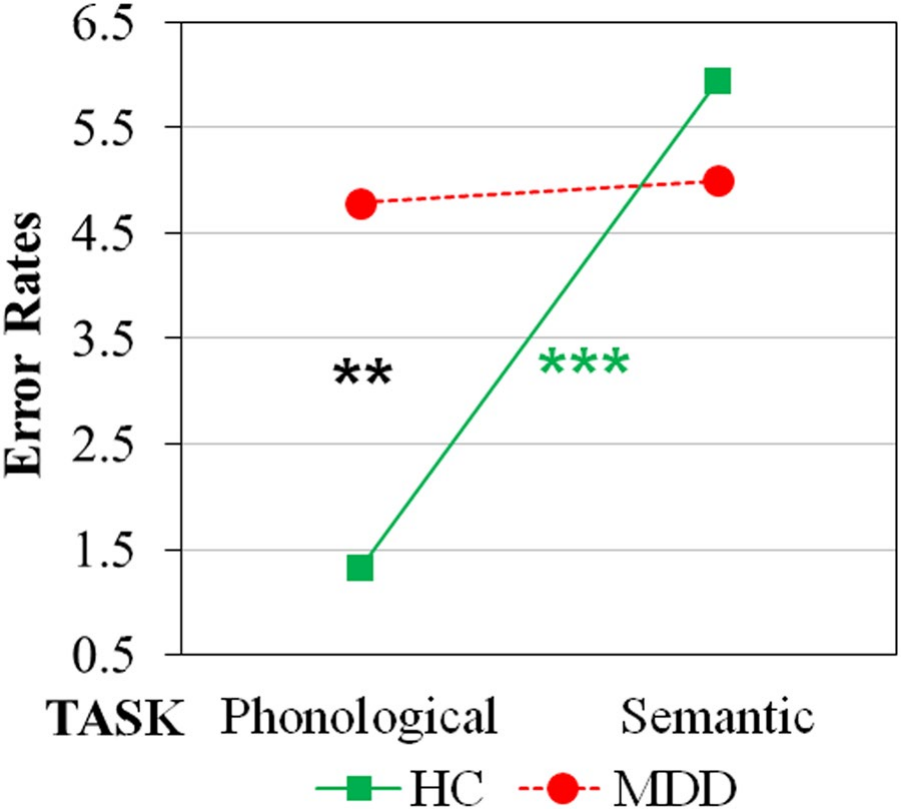
Error rate (ER) analysis revealed two-way Group by Task interaction. HC = healthy controls; MDD = major depressive disorder patients. Asterisks: significant (p < 0.05) post-hoc comparisons.

Analysis of ERs showed a significant main effect task factor [*F*(1,60) = 8.50, *p* < 0.01], with ERs being higher for the semantic task (5.47% ± 5.12%) than the phonological task (3.06% ± 4.26%). However, the two-way group-by-task interaction [*F*(1,60) = 7.09, *p* < 0.01] revealed significant post hoc differences only for healthy controls (Fig. 1).

Controls showed significantly higher error rates on the semantic task (5.94% ± 6.56%) than on the phonological task (1.33% ± 1.88%; *p* < 0.001), but depressive patients exhibited the same percentage of errors on all tasks (5.00% ± 2.95% and 4.79% ± 5.29%). Thus, patients made more errors than healthy controls on the phonological task only (*p* < 0.01).

### Electrophysiological data – alpha EEG band

**Figure 2:**
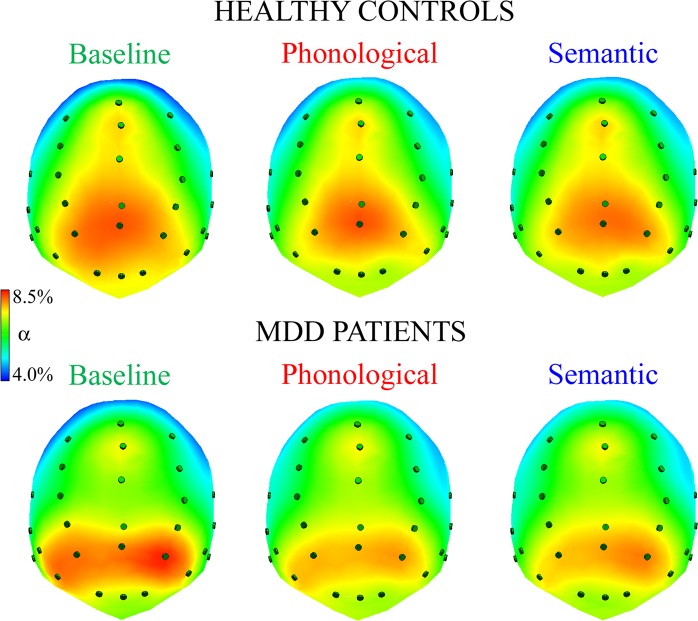
Spline maps of normalized alpha amplitudes during resting state, baseline condition (left panel), phonological and semantic linguistic tasks (middle and right panels, respectively) in HC and MDD patients. Qualitative analysis of spline maps suggests that, regardless of group, participants had bilateral alpha activity and greater levels in posterior regions, particularly in the baseline (control condition).

Figure 2 shows normalized alpha amplitude in healthy controls (top) and depressive patients (bottom) for each task: higher levels of amplitude are shown in red and lower ones in blue.

**Figure 3:**
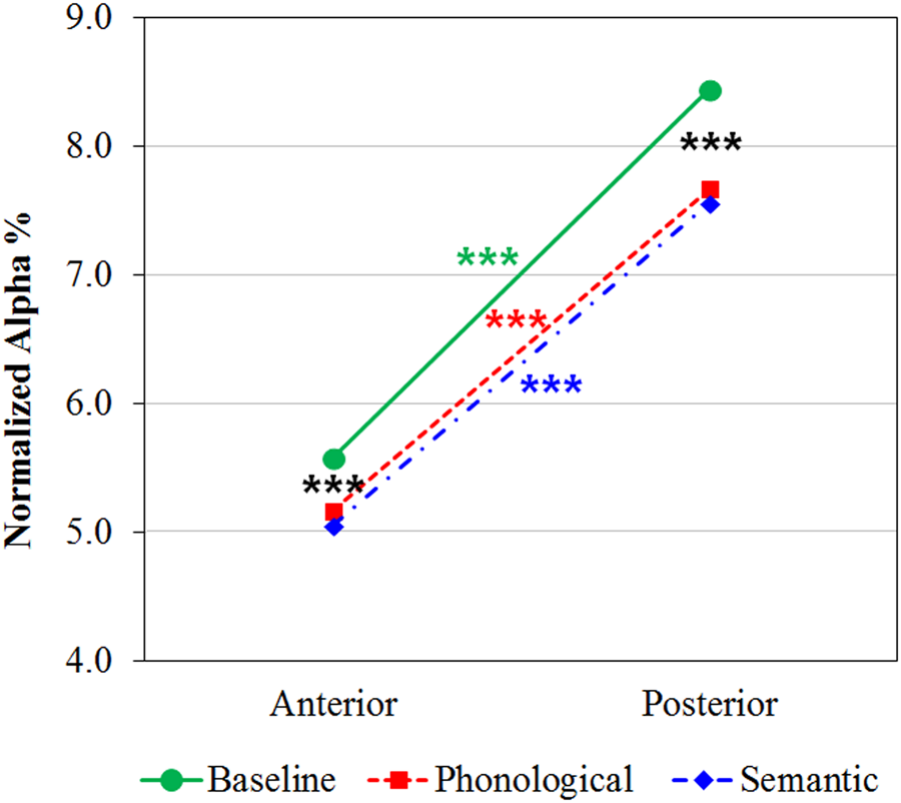
Alpha band analysis: significant two-way Task by Region interaction. Alpha distributions to resting state, baseline condition (green line), phonological (dotted red line) and semantic tasks (dotted-spotted blue line) are shown for anterior and posterior brain regions, regardless of group. Asterisks: significant (p < 0.05) post hoc comparisons.

Consistent with the pattern revealed by spline maps, the ANOVA on the normalized alpha EEG band showed a significant main effect of task factor [*F*(2,120) = 11.19, *p* < 0.001, GG *ε* = 0.90]; independent of group, a higher alpha level was found at baseline, compared with that elicited by both phonological and semantic tasks. In addition, the significant main effect of region factor [*F*(1,60) = 206.52, *p* < 0.001] showed higher alpha levels in posterior rather than anterior sites. However, the significant two-way task-by-region interaction [*F*(2,120) = 5.64, *p* < 0.01, GG *ε* = 0.85] revealed that this pattern of greater posterior than anterior alpha amplitude characterized all tasks (all *p*s < 0.001), but it was significantly greater in both anterior and posterior sites in the baseline condition, compared with both linguistic tasks (all *p*s < 0.001; Fig. 3).

No significant differences were found between alpha distribution in phonological and semantic task, nor was there an interaction with group factor.

### Electrophysiological data – beta EEG band

**Figure 4:**
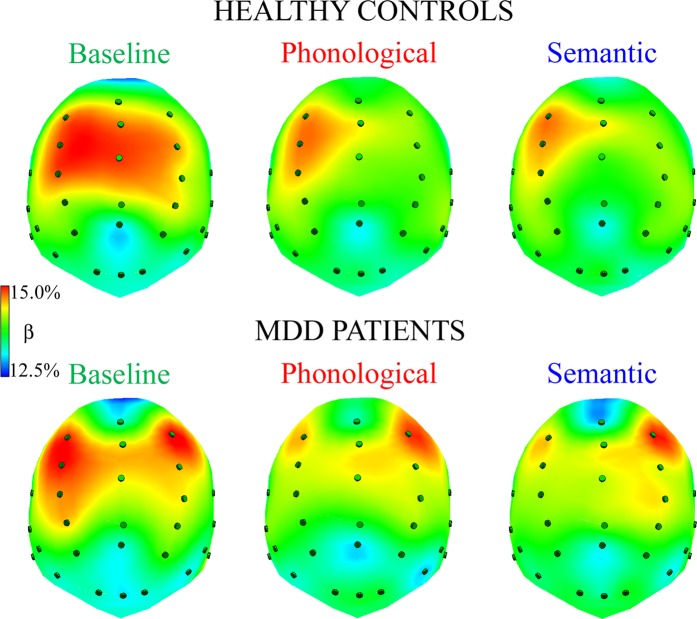
Spline maps of normalized beta amplitudes during resting state, baseline condition (left panel), phonological and semantic linguistic tasks (middle and right panels, respectively) for HC and MDD patients. Qualitative analysis of spline maps suggests that, compared with controls, depressed patients had lower levels of beta rhythm in anterior regions of the left hemisphere during the two linguistic tasks. In addition, controls showed an anterior, left-lateralized pattern of activation (i.e., higher percentages of beta band in anterior left vs. right regions) and a bilateral posterior activation in all tasks.

Figure 4 shows normalized beta amplitude in healthy controls (top) and depressed patients (bottom) for each task: higher levels of amplitude are shown in red and lower ones in blue.

**Figure 5:**
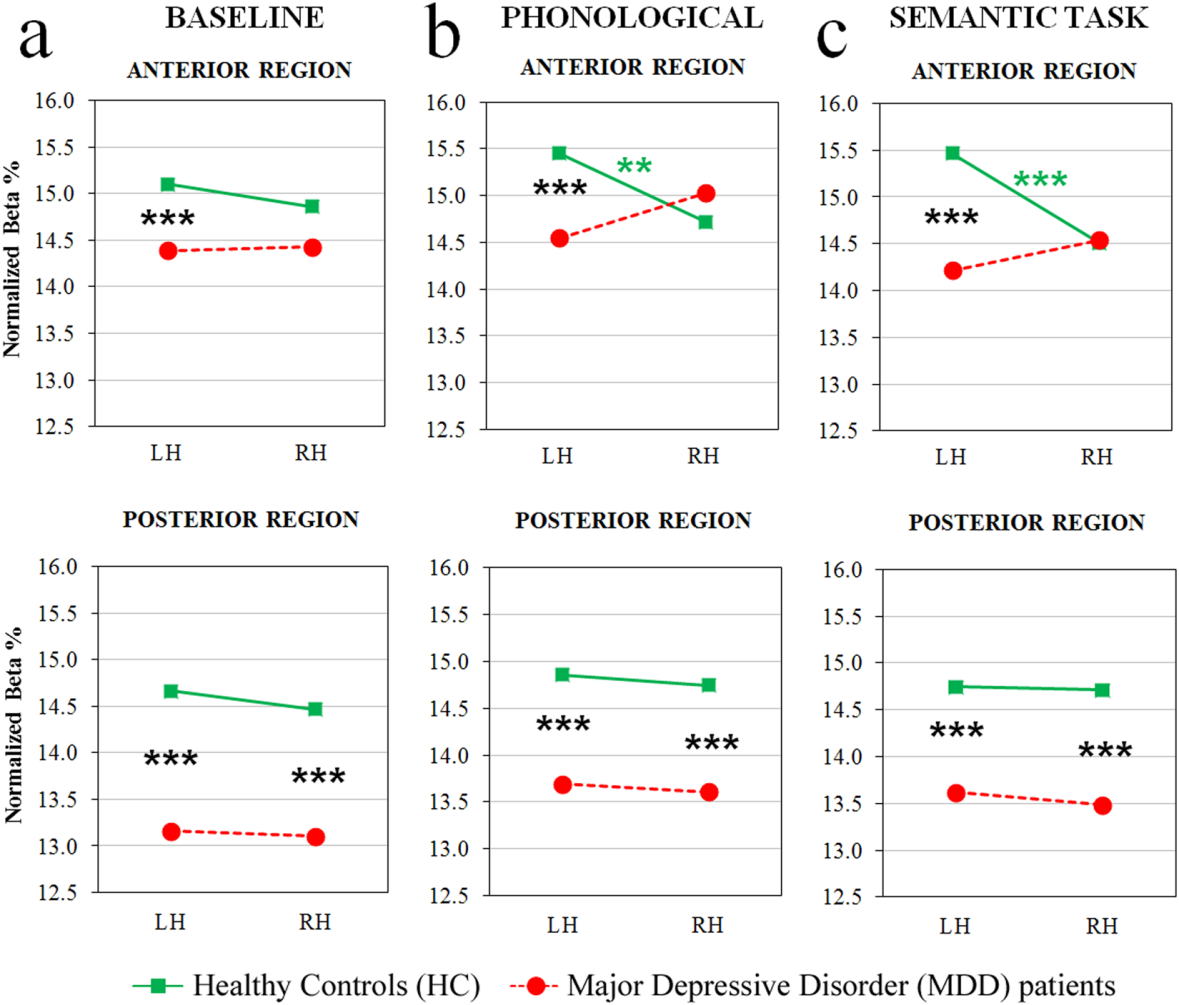
Beta band analysis: significant four-way Group by Task by Region by Laterality interaction. Beta distributions to resting state, baseline condition (left panels), phonological (middle panels) and semantic tasks (right panels) are shown for anterior (top row) and posterior brain regions (bottom row), for HC and MDD patient groups (green and red dotted lines, respectively). Asterisks: significant (p < 0.05) post-hoc comparisons.

The ANOVA on normalized beta amplitudes revealed a significant main effect of the region factor [*F*(1,60) = 14.64, *p* < 0.001], beta amplitude being greater in anterior vs. posterior clusters. In addition, the four-way interaction Group x Task by Region x Laterality [*F*(2,120) = 4.79, *p* < 0.01, GG ε = 0.95] showed that beta amplitude exhibited specific patterns of activation, depending on group and task. Regarding anterior sites (Fig. 5, top panel), healthy controls exhibited a bilateral pattern of activation during the baseline condition, and significantly greater left vs. right beta amplitude during both phonological and semantic tasks (all *p*s < 0.001), the amplitude of left clusters being significantly increased on both linguistic tasks with respect to the baseline (all *p*s < 0.05). Conversely, depressed patients showed a bilateral pattern of activation in all tasks (Fig. 5, red dotted line), the amplitude of the right cluster being significantly increased on the phonological task, relative to both the baseline and the semantic task (all *p*s < 0.05). In addition, the between-group comparison showed a significant difference only in the left cluster of all conditions, depressed patients’ beta amplitude being significantly decreased, relative to that shown by healthy controls (all *p*s < 0.001).

Regarding posterior sites, both groups revealed a bilateral pattern of activation (Fig. 5, bottom panel), and depressed patients showed significantly lower beta amplitude than healthy controls (all *p*s < 0.001).

### Spearman’s correlations

This analysis, carried out on patients’ and controls’ data, provided essential information for interpreting depressed participants’ beta distribution within the left frontal clusters. Spearman’s correlations were computed between the scores achieved by patients on STAI-Y1, positive and negative affect from the PANAS, and the beta amplitudes obtained at left frontal sites during baseline and linguistic tasks.

In HC, no significant association between beta amplitudes and either anxiety levels or positive affect values (all *p*s > 0.05) were found, whereas MDD patients showed a significant positive correlation between the scores obtained for the PA-PANAS subscale and the anterior left beta amplitude in baseline (*r*(28) = 0.42, *p* = 0.02). In depressed patients, the smaller the cortical activation in left frontal sites during resting state baseline, the lower the positive affect scores.

## Discussion

The present study, using language as a very general biological marker probe^43^ to assess cortical alterations of the psychotic spectrum, tested the hypothesis of a hypoactivation of the left prefrontal cortex area as an endophenotype of increased risk to develop depression.

Concerning behavioral data, MDD patients showed, during task execution, response times (RTs) similar to those collected from healthy controls, suggesting that drug treatment did not alter patients’ motor skills or response readiness. This is consistent also with the view that this disorder does not necessarily interfere with responding, as our experimental paradigm and linguistic tasks are relatively undemanding (on average, error rates were lower than 6.5%, regardless of group). Considering error rates, no group difference was found during the semantic task which, for HC, is typically more difficult than the phonological one. Instead, depressed patients made more errors than healthy controls during the phonological task. This task is usually considered to be very easy by HC, and is typically associated with strong left frontal lateralization^43,44^. Thus, the relatively greater error rates on the phonological task represents a small, but specific linguistic impairment, which is related to the reduced activity found in the cortical area underlying this task (see further).

Regarding the electrophysiological results, we measured the typical frontal alpha and beta EEG asymmetry in resting state and during two linguistic tasks. In the two conditions, the main effect of the group factor was not significant; patients had no overall greater alpha nor decreased beta amplitudes, relative to healthy controls, an interesting result that points to the lack of collateral effects of pharmacological treatment on brain activity of MDD patients. This conclusion was led by two lines of evidence. First, patients and controls showed comparable alpha EEG waves: there is no plausible evidence supporting the hypothesis that drug treatment selectively alters beta EEG rhythm, without affecting alpha EEG. Second, Beta EEG activity measured at anterior right sites showed no significant group differences neither at rest, nor during linguistic tasks: thus, also considering beta rhythm, there was a cluster of four electrodes that showed similar amplitude in patients and controls, a result pointing to the idea that drug treatment did not affect EEG rhythms.

Of interest for our study, frontal alpha asymmetry in HC and MDD groups was similar during resting state, and essentially not lateralized. This is in line with the heterogeneous and contradictory results reported in a recent review of the empirical studies on alpha asymmetry as a predictor of MDD^17^ that concluded that, due to the large variance in patient samples, the overall effect size across all 16 quoted studies was negligible.

With respect to the frontal asymmetry measured during the linguistic task, we expected: a) that tasks, in general, force participants’ brains to recruit more stable neural networks; b) that beta EEG is a better index (as compared with alpha EEG) for measuring cortical activation in a non-resting condition; and c) that, in line with the evolutionistic model of Crow^41,42^, MDD patients display a language-related altered frontal asymmetry. As expected, controls showed a clear pattern of greater activation of the left frontal sites on the two linguistic tasks. This asymmetry was mainly frontal, and was related to the rationale of our paradigm, which stresses verbal working memory – a strategic choice useful for measuring hypofrontality in patients of the psychiatric triad^37,40^ Instead, on both linguistic tasks, MDD patients showed a pattern of bilateral activation, marked by significantly decreased cortical arousal of left frontal sites with respect to HC, revealing patients’ inability to recruit these regions to carry out the task. Indeed, in depressed patients, the significantly worse performance (compared with controls) on the phonological task was associated with reduced left frontal EEG activation. Thus, behavioral and physiological results were coherent, and may be related to the predominance of processes and neural networks that are competitive and alternative to the phonological processing, dominant over left frontal sites. Alternative networks located in other cortical sites, such as in the right frontal regions, may be associated with negative mood and the presence of ruminations typical of depressed patients. Prior research^45^ showed how direct stimulation of the right vs. left frontal regions with transcranial Direct Current Stimulation (tDCS) elicited greater rumination thoughts in healthy samples. Unfortunately, we did not measure ruminations with specific tools (i.e., questionnaires and clinical interviews) that would have allowed to address directly this interpretation, but we found a result that indirectly supports it. The positive correlation between positive affect (PA-PANAS) and left frontal beta activity and the negative correlation of PA-PANAS with depression is in line with the view that decreased cortical arousal at left frontal sites is related to reduced positive affect and the latter, in turn, is related to depression. Although the role of rumination remains to be investigated in more targeted future studies, the observed pattern of results suggests that physiological activation of the left frontal regions is a critical hub for eliciting positive emotions and feelings, which spares with the valence hypothesis of emotion, which postulated that the left hemisphere is specialized for positive emotions^46–49^.

The use of a linguistic paradigm seems an odd approach to studying a disorder, major depression, which is centered, in principle, on a deficit in the affective domain. However, after more than a century since the first psychiatrists suggested an unitary view of psychosis^50^, there has recently been a reprise of the idea that psychotic manifestations lie on a continuum^32^, going from MDD to BD to schizophrenia, depending on the severity of symptoms and cognitive impairment. These psychiatric disorders have different core symptoms, but all are characterized by a disruption of the integration among different brain regions^51^ and the underlying processes, together with an abnormal reinforcement of a few neural circuits^52^. Language is the most complex process emerging from our brain; it involves the largest cortical network^53^, and requires the cooperation and integration of the whole cortex^54^ and a precise hierarchy between and within the hemispheres^41,42^. Therefore, language networks can be very sensitive to any perturbation of this complex neuronal asset such as it occurs in the most severe psychiatric disorders. We used simple linguistic tasks to probe the altered hemispherical asymmetry in psychiatric patients. In line with this functional aim, the disorders of the mentioned psychiatric triad are often associated with a spectrum of cognitive impairments including symptoms related to linguistic and metalinguistic domains: delusions, thought disorders, ruminations, hallucinations, semantic confusion (DSM-5)^33^. In schizophrenia, the neurocognitive impairments that affect patients are mainly in the working memory and language-related domains^55^. Major depression has been found associated with a number of cognitive deficits^22^ which include working memory^27^, executive functions^25^, but also semantic fluency, visuospatial backward and digit span forward tests^26^. Independently from the cognition field, a substantial number of studies has shown a clear altered frontal asymmetry at rest in depressed patients or associated with depressive symptoms^3,8,11,12^. Particularly, depressive symptoms and mood disorders have been related to a hypoactivation of left prefrontal areas. Therefore, there is increasing converging evidence from different fields on the hierarchical leading role of left prefrontal cortex over the right homologue and the left posterior ones, in mood, higher-level cognition and language. Neurologic patients with lesions at this level tend to reorganize the whole linguistic network also in posterior areas distant from the lesion site^56^ and, importantly, aphasic patients report with high frequency the onset of depression which is maximal when lesion involves left prefrontal sites very close to Broca’s area^57^. Recent research found that Broca’s area – together with its role in articulation and phonological encoding^58,59^ – is also involved in hierarchical as well as metalinguistic organization of higher linguistic processes (for review, see^60,61^). Furthermore, this area also plays a critical role within the left hemisphere, by leading high-order linguistic organization (e.g., thinking, action planning, and goal-directed behaviors)^62^. In the present study, the reduced activation found in the left frontal cortex fits well with the greater error rates committed by MD patients, compared to controls, during the phonological task.

In terms of further evidence of the link between language and psychosis, using the same linguistic paradigm, we found consistent evidence of a lack of frontal asymmetry (i.e., left > right activation in HC) during linguistic tasks in schizophrenia patients^37,38,40^ also results in agreement with the evolutionary hypothesis of T. J. Crow^41,42^ on the key role of altered language asymmetry in the origins of psychosis.

## Limitations and conclusions

Left frontal regions, especially Broca’s area, appears to represent a fundamental hub capable of organizing language and mood, but also high-level human processes that are related more indirectly to language, such as thinking, action planning, and goal-directed behavior. MD patients showed a reduced left prefrontal activation during linguist tasks that was not observed in resting state; we also found a behavioral impairment specifically to the phonological task that, from the literature, has its main hub in left Broca’s area. The correlation found with positive affect evidenced how patients with the lowest score in this scale had also the lowest activity at left prefrontal sites during rest. Given the central hierarchical role of this area over the right frontal homologue, as well as over the left temporo-parietal regions^56^, our results contribute to current research by showing how the most severe psychiatric disorders, although characterized by a variety of symptoms, some of which apparently very different, had in common a dysfunction of this complex region that regulates a number of processes essential for healthy human mental functioning. The merit of our study was to situate major depression in a larger framework that includes all major psychiatric disorders (especially schizophrenia, relatively more investigated than bipolar disorder) in an evolutionistic perspective, showing their relationship with language alteration, the unavoidable association of such alteration with human cognition and behavior, and the key role of the left frontal area in psychopathology.

For its many pioneering features, this study was unable to address many issues related to symptom severity and behavioral and cognitive correlates of major depression; many issues can be addressed in future studies by reducing the phenotypic variability of the patients through their splitting according to several variables, including disease duration, depression severity, symptoms (e.g., ruminations), and extent of cognitive impairment.

## Methods

### Participants

The psychiatric group consisted of 30 MDD patients (22 women, 8 men; mean age ± SD: 53.50 ± 11.62 years; mean years of education ± SD: 11.67 ± 3.90 years) recruited from the *Psychiatric Unit* of *Mood Disorders - Psychiatric Clinic, Neuroscience Department, University of Padova (Italy)* and the *Psychiatric Unit* of *Bussolengo Hospital* (ULSS 9, Italy) according to the following criteria: all patients were right-handed, according to the Edinburgh Handedness Inventory^63^; they had been diagnosed as major depressive patients during the acute phase, on the basis of the symptoms exhibited for more than 6 months, according to DSM-IV-R criteria; and at the time of the present study, all patients were in a euthymic state. The diagnosis, ascertained by the psychiatrists of the wards, who administered the *Structured Clinical Interview for DSM Disorders* (SCID), classified all patients as suffering from major depressive disorder (further details on clinical features and drug treatment of MDD patients in Supplemental Materials). In addition, prior to the experimental session, depressive patients were tested with: Hamilton Rating Scale for Depression (HAM-D)^64^, Beck Depression Inventory-II (BDI-II)^65^, State-Trait Anxiety Inventory-Y1 (STAI-Y1, State version)^66^. We also administered the Positive And Negative Affective Scale (PANAS) test, aimed at measuring the level of positive and negative affect – a dimension sensitive to mood deflection^67^.

The control group consisted of 32 right-handed healthy volunteers (21 women, 11 men; χ^2^(1) = 0.43, *ns*), matched for age (mean ± SD: 52.83 ± 11.90 years; *t*(60) = −0.22, *ns*) to the patient group. The STAI-Y1 and the PANAS test were also administered to this group a few minutes before the beginning of the EEG session.

All subjects gave their written informed consent to participation in this study, which was approved by the Ethics Committees for the Psychological Research of the School of Psychology and the School of Medicine (Protocol number 1240), University of Padova, and performed in accordance with the ethical standards laid down in the Declaration of Helsinki.

### Stimuli, tasks, and procedure

Stimuli consisted of bisyllabic or polysyllabic Italian content words, selected from a frequency dictionary of 5000 written Italian words^68^. Words were presented in pairs on a 17” computer monitor, one at a time, with an inter-stimulus interval of 2 s: the first word (W1) remained on the screen for 1 s and the second word (W2, or target) remained until the subject responded by pressing a keyboard button, but in no case longer than 5 s^43^. Word pairs were administered in two separate blocks, which corresponded to two linguistic tasks; thus, the same words were presented as W1, but in different randomized order across tasks. Upon W2-target presentation, participants had to decide whether word pairs rhymed (phonological task), and whether target word W2 was of the same category as W1 (semantic task; for further details, see^43^). Each task included 80 trials/word-pairs, 50% matches being randomly interspersed with 50% mismatch trials; task order was randomly varied across participants.

The experimental session began with a 5-min, open-eyes resting state EEG, recorded while participants were seated, relaxed, and had their eyes open (baseline condition).

### Data recording and analysis

The mean performance of behavioral measures (ERs and RTs) was compared between groups and among tasks.

Electrophysiological activity was recorded with 38 tin electrodes mounted on an elastic cap (ElectroCap) and positioned according to the International 10–20 system^69^. All cortical sites were referred online to Cz, and re-referenced off-line to the mean activity of the whole scalp by the average reference procedure. The sampling rate was set at 500 Hz, and impedance was kept below 5 KΩ. After data collection, EEG signals were corrected for blinking and eye movement artifacts, according to the eye movement modeling approach of Ille *et al*.^70^ All EEG data were divided into 2048-ms time intervals: indeed, given the constraint of BESA software to use 2^n^ samples, we needed to force the width of each interval to 1024 samples, corresponding to a 2048-ms interval. Each EEG task was divided into 2048-ms time intervals following W1 presentation, i.e., after the first word (from the same list for both tasks) offset, and corresponding to the whole CNV 2-sec interval, in which no stimuli were shown on the screen. Thus, every task included 80 samples with 0.488 Hz FFT resolution. For this reason, we also divided the Baseline EEG epoch (control condition, with no active task) into 80 2048-ms time intervals. Artifact rejection procedure was performed during each interval, with both amplitude and derivative thresholds (with respect to time) (250 μV and 100 μV/ms, respectively). The remaining epochs were also visually inspected in order to remove any residual artifacts, as well as trials corresponding to response errors: on average, 88.60% of the epochs were accepted, all corresponding to behaviorally correct responses, equally distributed among groups and tasks (range: 48/80 [60%] to 80/80 [100%] trials). After windowing each interval with a tapered cosine, the FFT was averaged across those epochs that were finally free of residual artifacts. In the following step, EEG amplitude was normalized within each electrode as the contribution of each band to the whole 0.488–100 Hz spectral range, and expressed as a percentage. Normalization allowed us to quantify the relative contribution of each EEG band with respect to total spectral power (% value) in the two main groups (healthy controls vs. depressive patients) and to compare the same scalp locations in all samples.

For statistical purposes, the amplitudes of alpha (8–12 Hz, effective α range 8.30–11.71 Hz) and high-beta (in the text indicated for simplicity as beta only) bands (20–35 Hz, effective β range 20.50–35.14 Hz)^71^ were examined. The electrodes were grouped into four clusters with two spatial factors consisting of two levels each: anterior-posterior asymmetry and laterality^38,40,43^. Each quadrant therefore included the average amplitude of 4 electrodes: Anterior Left (AL: F9-F7-F3-FC3), Anterior Right (AR: F10-F8-F4-FC4), Posterior Left (PL: P3-P7-TP7-O1), and Posterior Right (PR: P4-P8-TP8-O2).

Considering demographic and anxiety/mood scales, separated between groups, Student’s *t* tests were carried out on age, education, handedness, STAI-Y1 and PANAS subscales, whereas gender distribution was analyzed using the non-parametric *χ*^2^ test. In addition, for the patient group only, Spearman’s correlation was carried out between HAM-D and BDI-2 scores and STAI-Y1 and Positive and Negative PANAS subscale values, in order to ascertain whether the (relative) severity of MDD symptoms were significantly linked with anxiety (STAI-Y1) as well as positive and negative affect (PANAS subscales) levels. Positive correlations were expected between patients with higher scores on both clinical scales (HAM-D and BDI-2) and higher anxiety and/or negative affect levels, whereas negative correlations were limited to significant associations with positive affect levels.

With regard to behavioral measures (mean ERs and RTs), ANOVAs included the between-subjects factor group (two levels: Controls vs. Patients) and within-subjects factor task (two levels: phonological vs. semantic task).

Separate ANOVAs were carried out for each EEG band (alpha and beta amplitudes), including the between-subjects factor group (two levels: controls vs. patients), and three within-subject factors: task (three levels: baseline vs. phonological vs. semantic), region (two levels: anterior vs. posterior) and laterality (two levels: left vs. right hemisphere). Tukey’s Honestly Significant Difference (HSD) test was used to make post hoc comparisons (*p* < 0.05) and the Greenhouse-Geisser correction was applied when necessary, that is, when variables with more than two levels were involved^38,40,43,71^.

In addition, as we expected a significantly reduced frontal activation in left hemisphere sites, Spearman’s correlation analyses were carried out between selected scales that showed different levels between HC and MDD patients, i.e., STAI-Y1 and Positive PANAS subscale (see *Patients’ overview* in the results section) and beta amplitudes obtained at left frontal ROIs during baseline and both phonological and semantic tasks, separate for HC and MDD patients, to ascertain whether reduced frontal activation in left brain regions represented the physiological correlate significantly linked to anxiety/mood modulation along the continuum ranging from health to MDD. Positive and negative correlations marked those individuals with higher scores on STAI-Y1 and PANAS subscales indices, and a higher or lower (respectively) beta percentage in left frontal ROIs.

## Data Availability

The data that support the findings of this study are available on request from the corresponding author. The data are not publicly available, due to privacy or ethical restrictions.
